# Acute lung inflammation and ventilator-induced lung injury caused by ATP via the P2Y receptors: an experimental study

**DOI:** 10.1186/1465-9921-9-79

**Published:** 2008-12-13

**Authors:** Hiroki Matsuyama, Fumimasa Amaya, Soshi Hashimoto, Hiroshi Ueno, Satoru Beppu, Mitsuhiko Mizuta, Nobuaki Shime, Akitoshi Ishizaka, Satoru Hashimoto

**Affiliations:** 1 Department of Anesthesiology and Intensive Care, Kyoto Prefectural University of Medicine, Kyoto, Japan; 2 Pulmonary Division, Department of Medicine, Keio University School of Medicine, Tokyo, Japan

## Abstract

**Background:**

Extracellular adenosine 5'-triphosphate (ATP) is an endogenous signaling molecule involved in multiple biological phenomena, including inflammation. The effects of extracellular ATP in the lung have not been fully clarified. This study examined 1) the biological roles of extracellular ATP in the pathogenesis of lung inflammation and 2) the possibility of involvement of extracellular ATP in mechanical ventilation-induced lung injury.

**Methods:**

The effects of intratracheal ATP on lung permeability, edema or lung inflammation were assessed by measurements of the lung wet-to-dry weight ratio and lung permeability index, immunohistochemistry and expression of key cytokines by real-time polymerase chain reaction. The ATP concentration in broncho-alveolar lavage (BAL) fluid from mice mechanically ventilated was measured by luciferin-luciferase assay. The suppressive effects of a P2 receptor antagonist on ventilator-induced lung inflammation were also examined.

**Results:**

ATP induced inflammatory reactions in the lung mainly via the ATP-P2Y receptor system. These reactions were alleviated by the co-administration of a specific P2 receptor antagonist. Mechanical ventilation with a large tidal volume caused lung inflammation and increased the ATP concentration in BAL fluid. P2 receptor antagonism partially mitigated the inflammatory effects of large tidal volume ventilation.

**Conclusion:**

Our observations suggest that the ATP-P2Y receptor system is partially involved in the pathogenesis of ventilator-induced lung injury.

## Background

Acute lung injury and acute respiratory distress syndrome are major causes of acute respiratory failure, and are characterized by pulmonary edema, neutrophil infiltration with hemorrhage and increased production of inflammatory mediators [1]. Although mechanical ventilation is indispensable for the survival of critically ill patients presenting with acute lung injury (ALI)/acute respiratory distress syndrome (ARDS) [2], clinical trials have shown that improperly delivered mechanical ventilation may worsen or cause lung injury [3]. Lungs exposed to ineffective ventilator settings often develop diffuse alveolar injury [4], pulmonary edema [5] and activation of inflammatory cells [6]. The development of ventilator-induced lung injury (VILI) has been closely related to an increased production of pro-inflammatory cytokines [7], and to the leakage of inflammatory mediators into the systemic circulation [8]. Ventilation with a small tidal volume lowers the pulmonary and systemic concentrations of inflammatory mediators [9], and has beneficial effects in patients with ALI/ARDS [10], as well as in patients without lung disease undergoing mechanical ventilation [11].

Adenosine 5'-triphosphate (ATP), a nucleotide normally present in the cytoplasm, plays a prominent role in energy metabolism. Besides its intracellular role, extracellular ATP is involved in the regulation of several biological processes such as nociception [12], renal cell growth [13], and bone remodeling [14] via P2 purinergic receptors in the cell surface. Purinergic receptors are present in the lung [15], and the alveolar epithelial cells release ATP in response to various stimuli [16]. Bronchial hyper-responsiveness in asthmatic patients is triggered by intrinsic ATP, suggesting an important role played by ATP in the inflammation of the airways [17]. The purinergic system participates in the mechano-sensory functions of the urinary system [18,19] and of the pain- and stretch-sensing neurons [20]. Since mechanical stress causes the release of ATP by the lung epithelial cells [21], and since ATP stimulates the release of inflammatory cytokines by cultured macrophages, dendritic cells, or both [22-26], the purinergic system may be involved in the development of inflammatory reactions from mechanical stress in the lung.

To define the role played by extracellular ATP in the pathogenesis of lung inflammation due to mechanical ventilation, we 1) examined the effects of ATP exogenously instilled in the airways, 2) measured the concentrations of extracellular ATP in broncho-alveolar lavage (BAL) fluid after mechanical ventilation, 3) determined whether a purinergic receptor antagonist can alleviate the lung injury caused by mechanical ventilation, and 4) documented the expression of the P2Y_2 _and P2Y_4 _ATP receptors in lung tissue. Some of the results of these studies have been previously reported in the form of an abstract [27].

## Methods

### Biochemicals

Adenosine 5'-triphosphate (ATP), selective P2Xs, P2Y_2 _and P2Y_4 _antagonist pyridoxal-5'-phosphate-6-azophenyl-2', 4 '-disulfonic acid (PPADS), selective P2Y agonist uridine 5'-triphosphate (UTP) and selective P2X agonist α,β-methylene ATP (α,β-MeATP) were obtained from Sigma-Aldrich (St. Louis, MO).

### Animals

All experimental procedures and protocols were approved by the Animal Care Committee of the Kyoto Prefectural University of Medicine. The experiments included 308 male, specific, pathogen-free, 6- to 8-week-old Institute of Cancer Research mice (Japan S.L.C. Co. LTD., Shizuoka, Japan).

### ATP instillation

Under general anesthesia with inhaled sevoflurane, the mice were intubated with a 24 gauge, modified animal gavage needle (Popper & Sons, Inc., New Hyde Park, NY). First we performed a 6–48-h time course study and a 100–200-mM dose-response study to determine the proper response time and amount of ATP instillation. In some mice, 50 μl of 100 mM ATP was instilled into the left main bronchus via the needle. Other mice received a) a mixture of 100 mM ATP and 50 mM PPADS, b) 200 mM UTP, or c) 200 mM α,β-MeATP. Control mice received the same amount of saline. Mice recovered from the anesthesia within 1 min, were returned to their cages, and were provided with unrestricted food and water. They were allowed to survive for 60 min or 24 h, then sacrificed with deep sevoflurane anesthesia for further experiments.

### Wet-to-dry lung weight ratio

The lung wet-to-dry (W/D) weight ratio was used as an index of lung water accumulation after the instillation of ATP. To measure the total amount of lung water, the animals were dissected under deep sevoflurane anesthesia, and the lung weight was measured immediately after its excision (wet weight). The lung tissue was then dried in an oven at 60°C for 5 days and re-weighed as dry weight. The W/D weight ratio was calculated by dividing the wet by the dry weight as described previously [28].

### Permeability index

The permeability index, an index of alveolar epithelial and endothelial permeability [29], was calculated by injecting 100 μl containing 25 μg of human serum albumin intravenously, via a tail vein, 23 h after the instillation of ATP. The mice were anesthetized with sevoflurane 1 h after the injection, blood was sampled from the inferior vena cava, and BAL was twice performed with 0.5 ml of normal saline. To avoid the contamination of blood into BAL fluid, the catheter was inserted into the trachea and BAL was performed through the catheter. The total recovery volume of lavage fluid was regularly in the range from 0.8 to 0.9 ml in each mouse. The whole blood and BAL fluid were centrifuged at 1,000 g for 10 min at 4°C, to obtain plasma and cell-free BAL fluid. The plasma samples and the cell-free BAL fluid supernatant were kept at -80°C until further analysis. The concentration of human albumin in each solution was determined by enzyme-linked immunosorbent assay, using a human serum albumin kit (Cygnus Technologies, Southport, NC). The permeability index was calculated as the human albumin concentration in BAL fluid/plasma ratio × 1,000.

### Histological examinations

The mice were sacrificed 24 h after the instillation of ATP, and the left lung was excised, fixed with 4% paraformaldehyde for 6 h, embedded in paraffin, and sectioned in 4 μm thick slices, which were stained with hematoxylin and eosin. Immunohistochemical staining was also carried out to detect the distribution of P2Y_2 _and P2Y_4 _receptors in the lung of untreated mice. The lung sections were deparaffinized in toluene and hydrated by passage through decreasing concentrations of ethanol solutions. The antigen was activated by autoclave at 121°C for 15 min, immersed in 10 mM sodium citrate buffer followed by a 20-min cool-down, and incubated with rabbit anti-P2Y_2 _antibodies (1:300, AlphaGenix, Sioux Falls, SD) or rabbit anti-P2Y_4 _receptor antibodies (1:100, Biomol International, L.P., Plymouth Meeting, PA) at 4°C for 3 days. Staining was performed using the biotin-streptavidin technique and developed with diaminobenzidine. Counterstaining was performed with methyl green.

### BAL fluid analyses

The mice were sacrificed 24 h after the instillation of ATP, and the left lung was twice lavaged with 0.5 ml of saline. In all of the mice, the recovery volume was >0.8 ml. After centrifugation of the BAL fluid at 400 g for 10 min at 4°C, the cell pellets were resuspended in 1 ml of saline. The total number of cells in BAL fluid was counted with a hemocytometer. Cytospins were prepared from resuspended BAL fliud cells, using a Shandon Cytospin^® ^3 Cytocentrifuge (Shandon, Astmoore, UK). Cell differentials were counted on the slides stained with Diff-Quik (Sysmex, Kobe, Japan).

### Expression of cytokine mRNA

Quantitative real-time reverse transcription (RT) polymerase chain reaction (PCR) was performed to measure the relative levels of expression of lung inflammatory cytokine gene. Total RNA was extracted from the left lung homogenates, using the TRIzol^® ^reagent (Invitrogen, Carlsbad, CA) according to the manufacturer's recommendations. The RNA concentration was measured by spectrophotometry. First-strand cDNA was synthesized from total RNA using a SuperScript Platinum^® ^Two-Step q RT PCR reaction Kit (Invitrogen, Carlsbad, CA) as instructed by the manufacturer. PCR primers for target gene were purchased from Takara Bio Inc. (Otsu, Shiga, Japan). Relative mRNA levels were measured with a SYBER green detection system on an ABI 7300 Real-Time PCR system (Applied Biosystems, Foster City, CA). All samples were measured in triplicate. We measured the expression levels of macrophage inflammatory protein-2 (MIP-2), tumor necrosis factor-α (TNF-α), interleukin-6 (IL-6) and IL-1β. The relative amount of expression of each gene was calculated as a ratio compared with the reference gene, glyceraldehyde-3-phosphate dehydrogenase (GAPDH).

### Mechanical ventilation

The mice were anesthetized with inhaled sevoflurane and intraperitoneal injection of pentobarbital (Abbot Laboratories, North Chicago, IL), 50 mg/kg. A vertical midline cervical incision was used for cannulation of the trachea with a blunt 18-gauge endotracheal tube. Immediately after the cannulation, the mice were connected to a model 683 mechanical ventilator (Harvard Apparatus, South Natick, MA) for the delivery of lung injurious ventilation with a 40-ml/kg tidal volume, or to a HSE-Harvard Mini-Vent (Hugo Sachs Elektronik-Harvard Apparatus GmbH, March-Hugstetten, Germany) for room air ventilation with an 8-ml/kg tidal volume, for 60 min. Positive end-expiratory pressure was set at 0 cmH_2_O for large, and 3 cm H_2_O for small tidal volumes ventilation. We chose a 40-mg/kg tidal volume for injurious ventilation, since our preliminary study showed no significant change of lung W/D weight ratio by the ventilation with 10–20 ml/kg tidal volume (data not shown). The control group underwent tracheotomy only. In some mice, both lungs were excised for measurement of the W/D weight ratio and analysis of cytokine mRNA expression. Others were processed to measure the alveolar ATP concentration in BAL fluid. Some mice received 60 μl of either sterile saline or 50 mM PPADS into the lung, 60 min before the onset of mechanical ventilation.

### ATP assay in BAL fluid

Following mechanical ventilation, 1 ml of sterile saline was slowly instilled from the endotracheal tube, and BAL fluid was collected, centrifuged at 800 g for 10 min at 4°C to prevent cytolysis, and the supernatant was used for the ATP assay. ATP in BAL fluid was measured by a luciferin-luciferase assay (Toyo Ink Co., Tokyo, Japan). The relative light intensity was recorded in a Lumat LB9507 luminometer (Berthold Technologies GmbH & Co. KG, Wildbad, Germany).

### Statistical analyses

All data are presented as means ± SEM. Between-groups comparisons were made by one-way analysis of variance with the parametric Student-Newman-Keuls multiple comparison post-test or the non-parametric Kruskal-Wallis test with Dunn's multiple comparison post-test. Instat 3 software (GraphPad Software Inc. San Diego, CA) was used for all analyses. p values < 0.05 were considered statistically significant.

## Results

### Effect of ATP instillation on the lung water content

**Figure 1:**
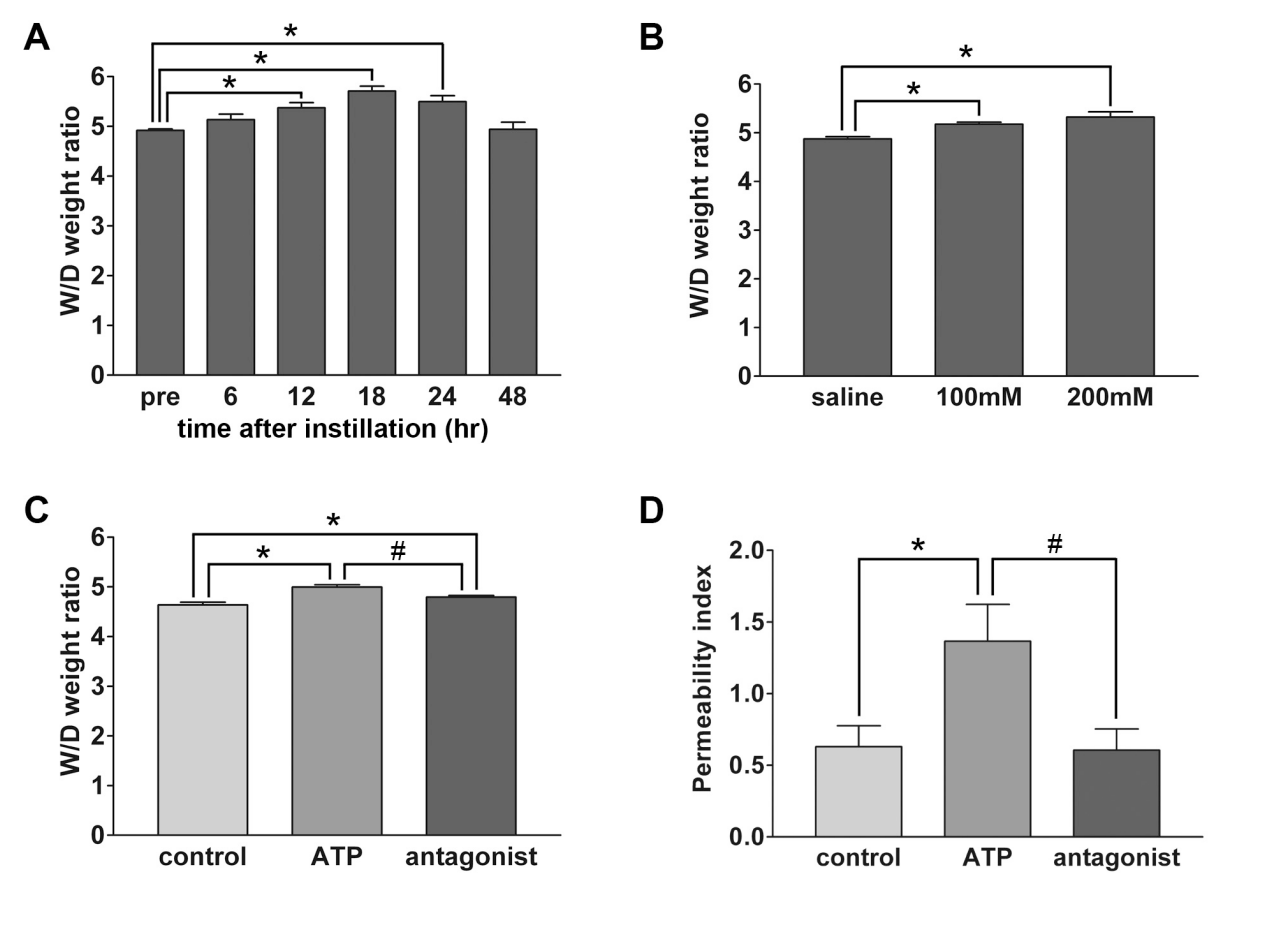
**ATP-induced lung edema and its mitigation by P2 receptor antagonist**. A. Time course of wet-to-dry (W/D) weight ratio following the instillation of ATP. 50 μl of 100 mM ATP was instilled into the left lung. The W/D weight ratio was calculated 6–48 h after the instillation. n = 5–7 mice in each group; *p < 0.05 vs. pre-treatment. B. Dose-dependency test. The W/D weight ratio was calculated 24 h after the 50 μl of 100–200 mM ATP instillation. The W/D weight ratio increased in a dose-dependent manner and the difference reached statistical significance at 100 mM of ATP. n = 11–15 mice in each group; *p < 0.05 vs. saline. C. Mitigation of ATP-induced lung water accumulation by P2 receptor antagonism. The increase of W/D weight ratio induced by instillation of 100 mM ATP was attenuated by the co-administration of 50 mM PPADS and 100 mM ATP. The W/D weight ratio was measured 24 h after the instillation. n = 5–9 mice in each group; *p < 0.05 vs. control; #p < 0.01 vs. ATP. D. Mitigation of ATP-induced lung permeability by P2 receptor antagonism. Increase in permeability of alveolar epithelial and endothelial cells by instillation of 100 mM ATP. This effect was inhibited by the co-administration of 50 mM PPADS and 100 mM ATP. The permeability index was calculated 24 hr after the instillation. n = 7 mice in each group, *p < 0.05 vs. control; #p < 0.05 vs. ATP.

Following the instillation of 50 μl of 100 mM ATP, the lung W/D weight ratio increased within 12 h after treatment, up to 24 h, and returned to baseline within 48 h (Figure 1A). Figure 1B shows the dose-dependent effects of ATP on the lung W/D weight ratio at 24 h following the instillation. In animals treated with >100 mM of ATP, the increase in W/D weight ratio was significant. In contrast, treatment with saline at a pH = 3.0 (similar to the ATP solution) did not increase the W/D weight ratio (data not shown).

In order to determine whether the effect of ATP was mediated by P2 purinoreceptors, we administered PPADS, a specific antagonist against the P2X and P2Y receptors [15], along with ATP. The simultaneous administration of PPADS, 50 mM, and ATP attenuated the increase in W/D weight ratio induced by ATP (Figure 1C).

The instillation, 24 h before the assay, of ATP, 100 mM, caused a significant increase in the albumin permeability index, a measure of the permeability of alveolar epithelial and endothelial cells (Figure 1D). The concomitant administration of PPADS and ATP inhibited the effects of ATP.

### ATP-induced inflammatory response in the lung

**Figure 2:**
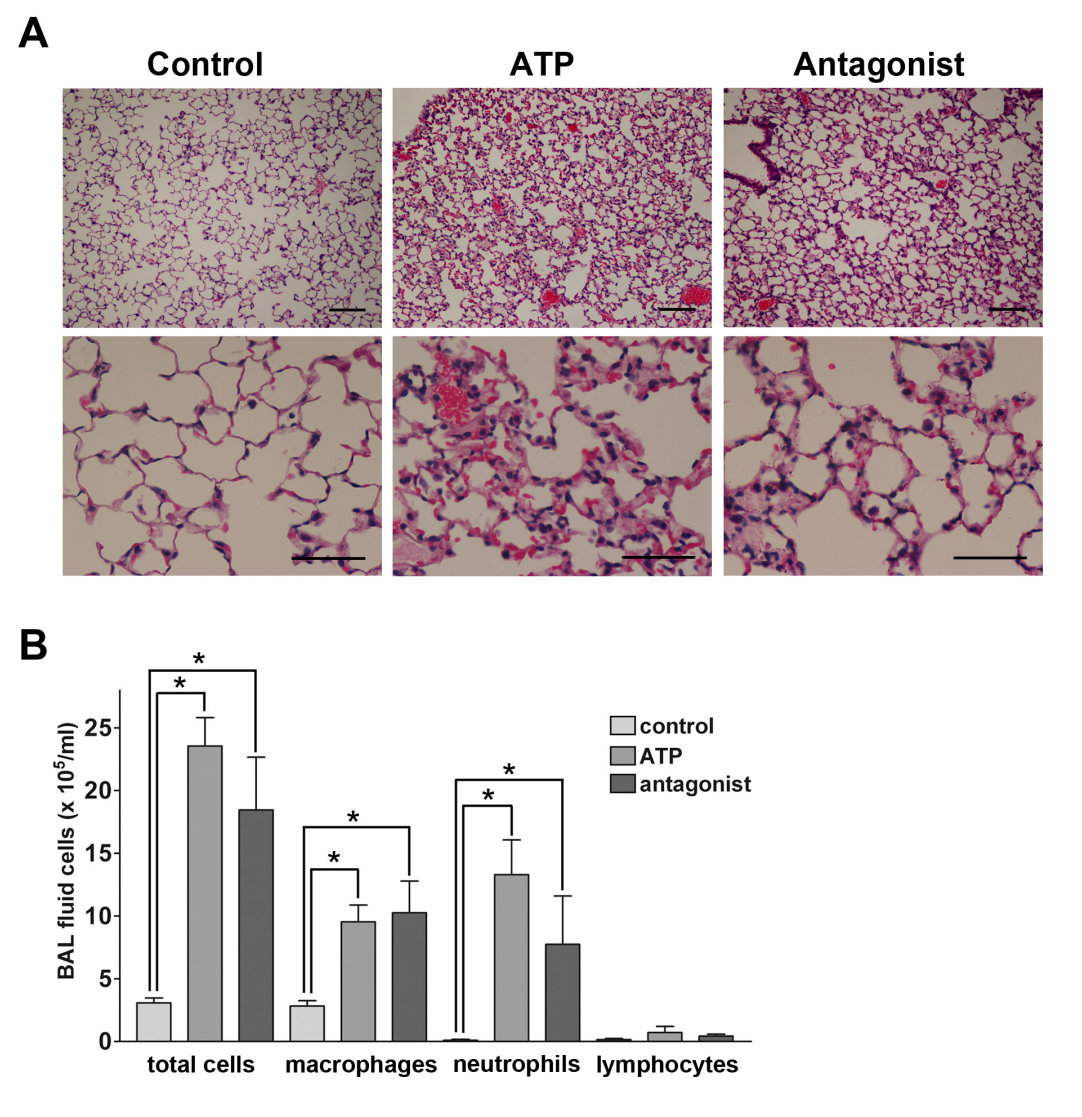
**Infiltration of inflammatory cells after instillation of ATP**. A. Hematoxilin-eosin staining of the lung. The instillation of ATP increased the thickness of the alveolar walls, and caused alveolar hemorrhages and infiltration of neutrophils and macrophages in 24 h. These histological changes were not enough mitigated by the simultaneous administration of PPADS. Bars = 100 μm in the upper panels, and = 50 μm in the lower panels. B. Total and differential cells count in BAL fluid after instillation of ATP. The instillation of 100 mM ATP caused significant increase in total cells, macrophages and neutrophils in BAL fluid in 24 h. n = 8 mice in each group; *p < 0.05 vs. control.

On histological examination, 24 h after the instillation of ATP, 100 mM, the thickness of the alveolar walls, the amount of alveolar hemorrhage and the numbers of neutrophils and macrophages infiltrating the lung were increased (Figure 2A). In contrast, the instillation of saline did not induce this inflammatory response in the control group. The histological derangement of alveolar architecture was partially mitigated by the simultaneous administration of PPADS and ATP. Therefore, the total number of cells in the BAL fluid was significantly increased in the mice instilled with 100 mM ATP 24 h before the assay. The differential cell counts showed that there were increased numbers of macrophages and neutrophils in ATP-treated mice (Figure 2B). The co-administration of PPADS limited the neutrophil infiltration, though the decrease was not statistically significant.

**Figure 3:**
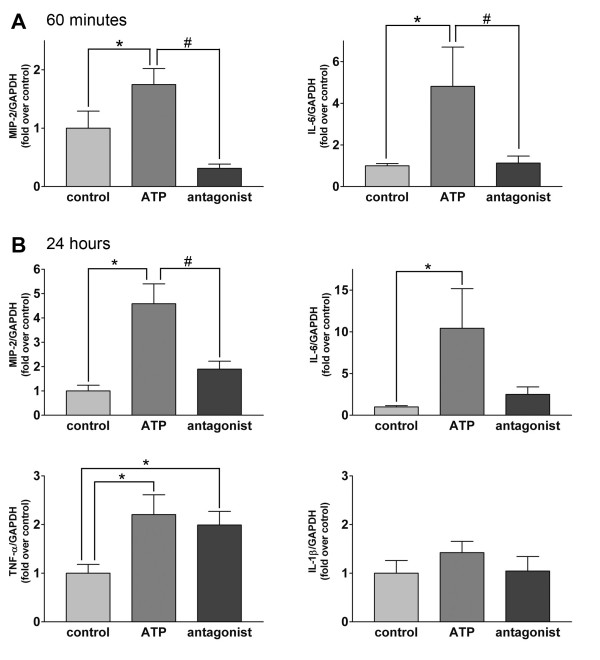
**Induction of expression of inflammatory cytokines by instillation of ATP**. A. Inflammatory cytokine expression in early phase. The instillation of ATP increased significantly the mRNA expression of MIP-2 and IL-6 in 60 min. PPADS limited the increase in the expression of these genes. n = 8 mice in each group; *p < 0.05 vs. control; #p < 0.05 vs. ATP. B. Inflammatory cytokine expression in 24 h. The instillation of ATP increased significantly the mRNA expression of MIP-2, IL-6 and TNF-α, but not of IL-1β. PPADS limited the increase in the expression of MIP-2 and IL-6 gene. n = 8 mice in each group; *p < 0.05 vs. control; #p < 0.01 vs. ATP.

Real-time PCR revealed that the induction of multiple inflammatory cytokines and chemokines is involved in the pulmonary inflammatory reaction induced by ATP. Exogenous ATP increase significantly the expression of MIP-2 and IL-6 mRNA within 60 min after its intratracheal instillation, and the simultaneous administration of PPADS and ATP inhibited the expression of these genes (Figure 3A). There was no significant change in the expression of TNF-α mRNA (data not shown). The instillation of ATP caused a significant increase in the expression of MIP-2, IL-6 and TNF-α mRNA within 24 h. The simultaneous administration of PPADS and ATP inhibited the induction of MIP-2 and expression of the IL-6 gene, however not that of TNF-α. The expression of IL-1β mRNA in the lung tissue, by contrast, was not modified by treatment with ATP (Figure 3B).

### Mediation of the effect of ATP on lung inflammation by the P2Y receptor

**Figure 4:**
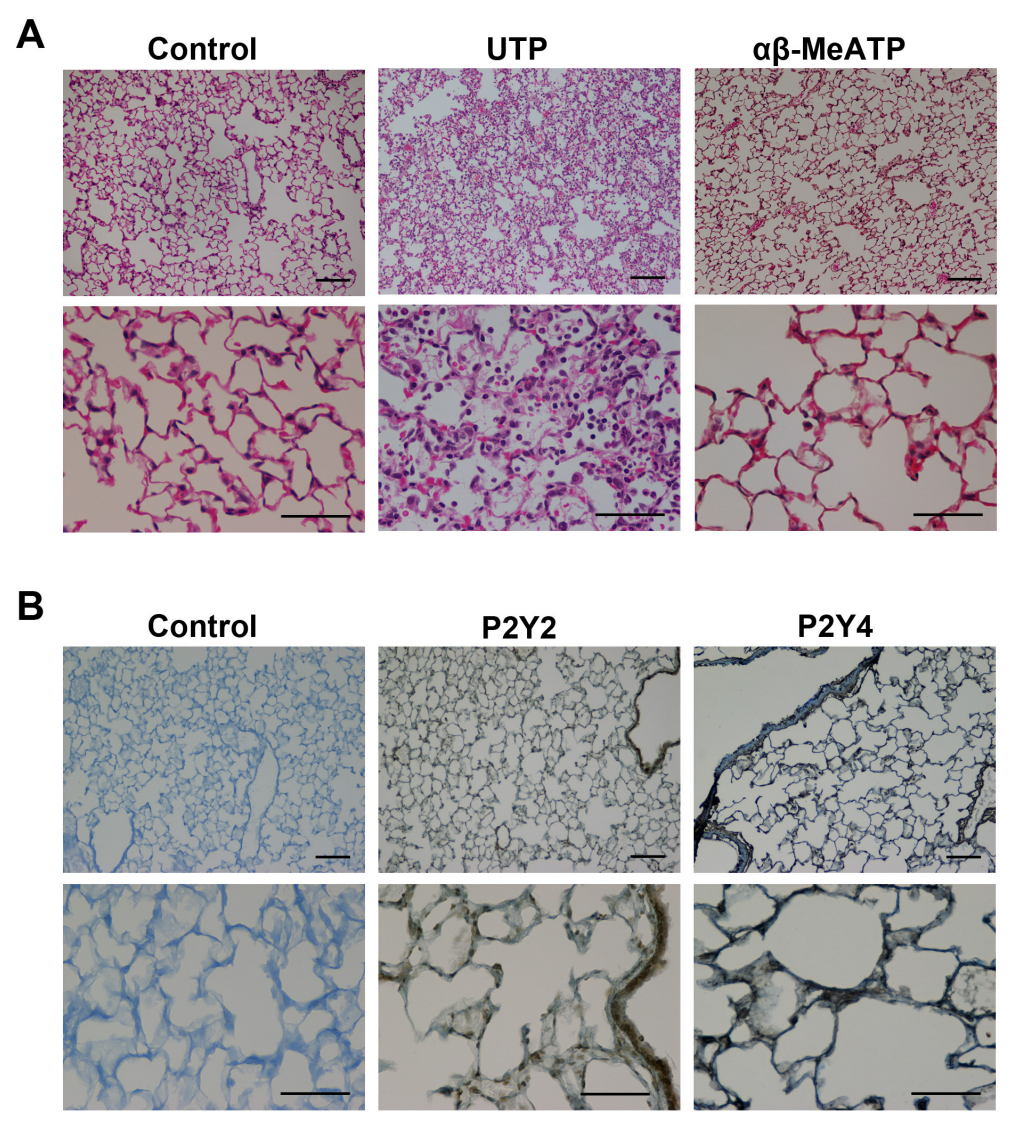
**Lung inflammation caused by P2Y and P2X selective agonist**. Hematoxilin-eosin staining of the lung 24 h after the instillation of UTP or α,β-MeATP. Infiltration of inflammatory cells, increased thickness of the alveolar walls and alveolar hemorrhages were observed in the 200 mM UTP-treated lungs. However, there was no evident derangement in the 200 mM α,β-MeATP-treated lungs. Bars = 100 μm in the upper panels, and = 50 μm in the lower panels. B. Immunohistochemistry of the P2Y_2 _and P2Y_4 _receptors. P2Y_2 _and P2Y_4 _receptors were detected in bronchiolar epithelial cells, alveolar walls and alveolar macrophages in the lung of untreated mice. Bars = 100 μm in the upper panels, and = 50 μm in the lower panels.

To identify the signal transduction of ATP-induced lung inflammation, we examined the effects caused by the instillation of UTP, an ATP analog selective for P2Y receptors, and α,β-MeATP, selective for P2X receptors, on the lung status. The instillation of UTP, 200 mM, caused a similar infiltration of inflammatory cells as that caused by ATP (Figure 4A). On the other hand, the instillation of α,β-MeATP caused no significant histological change in the lung. The instillation of UTP also induced a significant increase in W/D weight ratio (control; 4.781 ± 0.050, UTP; 5.065 ± 0.069, p = 0.0063) and permeability index (control; 0.4600 ± 0.0915, UTP; 0.8018 ± 0.2003, p = 0.0285).

Immunohistochemistry identified P2Y_2 _and P2Y_4 _receptor expressions in bronchiolar epithelial cells, alveolar walls and alveolar macrophages in the lung of untreated mice (Figure 4B).

### ATP secretion induced by large volume ventilation

**Figure 5:**
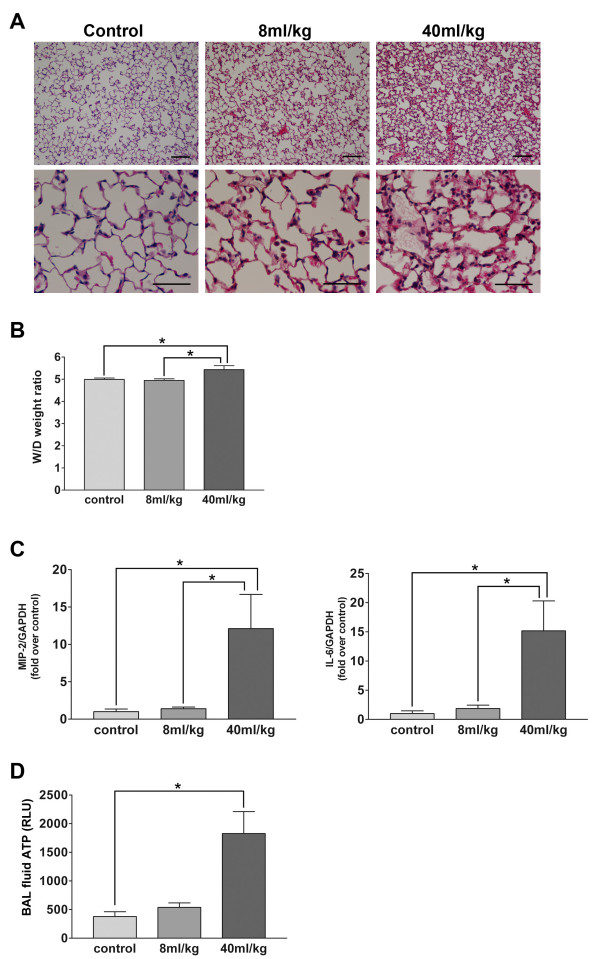
**Lung inflammation caused by large tidal volume mechanical ventilation**. **A**. Histological analysis showed a preserved lung parenchymal structure in the group ventilated with an 8-ml/kg tidal volume for 60 min. In contrast, an increased thickness of the alveolar wall and inflammatory cell invasion were observed in the group ventilated with 40-ml/kg tidal volume. Bars = 100 μm in the upper panels, and = 50 μm in the lower panels. **B**. Mechanical ventilation with a large tidal volume increased the lung W/D weight ratio significantly. This was not observed in the group mechanically ventilated with a small tidal volume. n = 8 mice in each group; *p < 0.05 vs. control and 8 ml/kg. **C**. The expression of MIP-2 and IL-6 gene was markedly increased in the lungs mechanically ventilated with a large tidal volume. n = 8 mice in each group; *p < 0.05 vs. control and 8 ml/kg. **D**. The ATP concentration in BAL fluid was significantly higher in mice ventilated with a large tidal volume than in non-ventilated control mice. n = 4–8 mice in each group; *P < 0.05 vs. control.

On histological examination, the thickness of the alveolar wall was increased and an invasion by inflammatory cells was observed in the lung of the group ventilated with a large tidal volume (Figure 5A). The lung W/D weight ratio increased significantly following 60 min of mechanical ventilation with the 40-ml/kg tidal volume, while no apparent change was observed in the group ventilated with the 8-ml/kg tidal volume (Figure 5B). The cytokine mRNA assay revealed a significant increase in the expression of MIP-2 and IL-6 mRNA that was limited to the lungs of mice ventilated with large tidal volumes (Figure 5C). These observations confirmed that mechanical ventilation with a large tidal volume constituted a suitable model of lung injury.

Compared to the spontaneously breathing control mice, the ATP concentration in BAL fluid, measured photometrically, was significantly increased in the animals exposed to the large tidal volume, but not those ventilated with a small tidal volume (Figure 5D).

### Mitigation of the ventilation-induced pulmonary inflammatory response by PPADS

**Figure 6:**
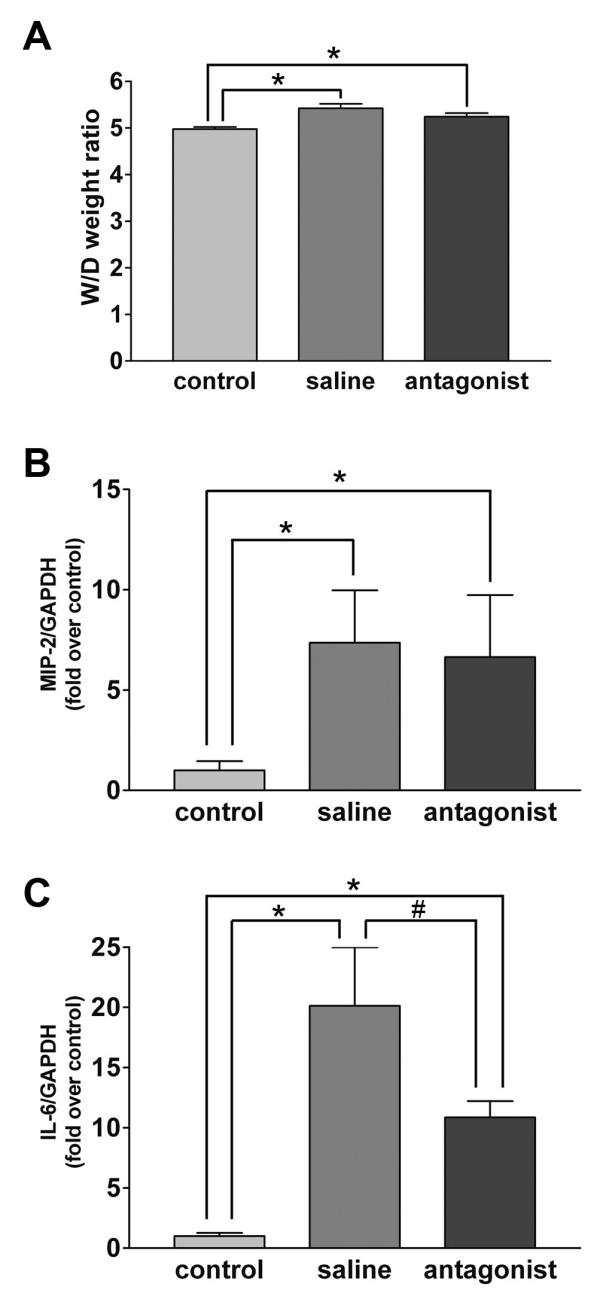
**Effects of P2 receptor antagonist on lung inflammation caused by large tidal volume mechanical ventilation**. **A**. The pre-instillation, 60 min before the onset of mechanical ventilation, of PPADS (60 μl of 50 mM) attenuated the increase in W/D weight ratio caused by large tidal volume mechanical ventilation for 60 min, though the difference was not statistically significant. n = 16 mice in each group; *p < 0.05 vs. control. **B**. Pre-treatment with PPADS did not significantly inhibit the induction of MIP-2 mRNA following large tidal volume ventilation. n = 8 mice in each group; *p < 0.05 vs. control. **C**. Pre-treatment with PPADS inhibited the induction of IL-6 following large tidal volume ventilation. n = 8 mice in each group; *p < 0.05 vs. control; #p < 0.05 vs. saline.

To confirm the involvement of the purinergic system in VILI, we instilled 60 μl of 50 mM PPADS or saline before the onset of mechanical ventilation. This administration of PPADS caused a significant blockade of the expression of IL-6 in the lung (Figure 6C), though did not limit the increase in the W/D weight ratio (Figure 6A), expression of MIP-2 (Figure 6B) and permeability index (data not shown).

## Discussion

ATP is believed to act in the intercellular signal transduction as a "purinergic system" in multiple organs, and is involved as an energy source in cellular metabolism. In the present study, exogenously applied ATP caused an inflammatory reaction by activating the P2Y purinergic receptors. The extracellular concentrations of ATP in the alveolar space increased as a result of the injury inflicted by mechanical ventilation with a large tidal volume, suggesting an important role played by the purinergic system in the development of mechanical VILI.

### Facilitation of lung inflammation by extracellular ATP

Following the intratracheal instillation of exogenous ATP, we observed increases in the W/D weight ratio and permeability index. Since the W/D weight ratio and the permeability index reflect, respectively, the lung water content and the vascular permeability status, our observations indicate that vascular permeability and lung water content, both manifestations of lung inflammation, were both increased. In the range that we tested, the action of ATP was dose-dependent, beginning 6 h after the instillation and persisting for up to 24 h thereafter. This was associated, histologically, with the aggregation of inflammatory cells in the alveolar tissue. These changes are attributed to a biological effect of ATP mediated by specific receptors, rather than to chemical or physiological effects exerted by the ATP solution, since administration of pH-adjusted saline had no effect on the lung edema or alveolar histology. PPADS alleviated ATP-induced lung edema and cytokine expression but it failed to inhibit the accumulation of inflammatory cells. PPADS is a purinergic receptor antagonist that acts on P2Y_2 _and P2Y_4 _[15], while extracellular ATP has been observed to induce chemotaxis of microglia, which acts as an innate immune system in the central nervous system, mediated by the P2X_4 _and P2Y_12 _receptors [30]. Thus, the differential effect of ATP might reflect a heterogeneous receptor signaling of ATP-mediated lung inflammation. While the activation of immune cell and consequent lung injury is mediated by P2Y_2 _and P2Y_4_, the migration of inflammatory cells is mediated by other purinergic receptors.

Previous *in vitro *studies have shown that extracellular ATP stimulates monocytes/macrophages or dendritic cells to release inflammatory cytokines, such as IL-1β [22,24-26] and TNF-α [23]. Our PCR study revealed that ATP increased the expression levels of IL-6, TNF-α and MIP-2, but not of IL-1β mRNA. This absence of increase in IL-1β mRNA level following ATP treatment is consistent with previous reports of a post-transcriptional regulation of IL-1β by ATP [22,31]. In addition to its pro-inflammatory activity, ATP regulates the status of fluids in lung tissue, stimulating the release of mucin and surfactant from bronchial epithelial and type II alveolar epithelial cells [32,33]. Therefore, the increase in lung edema that followed the instillation of ATP was the mixed consequence of an inflammatory reaction and a derangement of fluid exchange, both of which are due to the direct action of extracellular ATP.

### Involvement of the P2Y receptor in ATP-induced lung inflammation

The two subtypes of the purinergic receptor family are P2X, which is coupled with the ion channel, and P2Y, which activates the intracellular G-protein. To identify the receptor primarily involved in ATP-induced lung injury, we used the ATP analogue, α,β-MeATP, which acts selectively against the P2X receptor, and UTP, which acts selectively against the P2Y receptor [34]. While UTP induced an inflammatory response similar to ATP, α,β-MeATP had no apparent effect on the lung, suggesting that the activation of the P2Y receptor system was sufficient to promote lung injury. Among several subtypes of P2Y receptors, P2Y_2 _and P2Y_4 _are the most abundant in lung tissue extracts [15] and are expressed on alveolar macrophages in BAL fluid [35]. Our immunohistochemical analysis identified the expression of P2Y_2 _and P2Y_4 _in bronchiolar and alveolar epithelial cells and alveolar macrophages, which are both believed to be sources of inflammatory cytokines during acute lung injury [36,37]. Consistent with these observations, PPADS, a selective purinergic antagonist against P2X, P2Y_2 _and P2Y_4_, mitigated the inflammatory effects of the instillation of ATP. Therefore, ATP might activate epithelial cells, macrophages or both via P2Y_2 _and P2Y_4 _receptors to promote the production of inflammatory cytokines associated with lung injury.

### Involvement of ATP in mechanical lung injury

Worsening or induction of acute lung injury by mechanical ventilation is known as "VILI, and also as "ventilator-associated" lung injury. VILI is characterized by an increased alveolar permeability, pulmonary edema, infiltration of neutrophils, and the release of inflammatory mediators [38,39]. An increased cytokine expression accompanied by migration of inflammatory cells was observed in lungs ventilated with large tidal volumes. The concentration of ATP in BAL fluid was markedly increased under these circumstances, consistent with the release of ATP in response to alveolar epithelial cell stretch *in vitro *[40], or the increase in ATP or purine concentrations in BAL fluid after mechanical ventilation *in vivo *[41-43]. The concentration of ATP did not increase after lung protective ventilation, suggesting an essential role of ATP in mediating VILI. Alveolar epithelial cells or macrophages can produce pro-inflammatory cytokines such as IL-6, IL-8 and TNF-α when stretched *in vitro *[44-47] and promote VILI [48,49]. Since the instillation of ATP induced proinflammatory cytokines, ATP-P2Y signaling might act as a biological sensor that translates mechanical stimuli into production of cytokines. Yoshikawa et al. have shown that lung edema induces VILI independently [50]. Since exogenous ATP directly controls the fluid status in the lung, the lung edema caused by ATP might be another mechanism of VILI.

The antagonism of ATP-P2Y signaling by PPADS blocked the production of IL-6 induced by mechanical ventilation, illustrating the, at least partial, involvement of ATP signaling in the mechano-transduction and pathophysiology of VILI. PPADS prevented neither the production of MIP-2, nor the changes in W/D weight ratio and permeability index following ventilation. This might reflect the complexity of pathogenesis of VILI, even in ATP signaling.

In contrast to our observations, a recent study found that intravenous ATP enhanced endothelial integrity and alleviated LPS-induced lung injury in mice [51]. *In vitro *studies have shown that the biological effects of ATP are multiple, including monocyte chemotaxis [52] and enhanced endothelial integrity [53]. Discrepancies between our observations and those made by Kolosova et al. are probably attributable to a multimodal effect of ATP. We injected ATP intratracheally, which might have had a direct effect on the alveolar tissue. The limited efficacy of PPADS in the prevention of mechanical lung injury is, therefore, likely to reflect multiple and site-specific biological effects of ATP.

We found, in this study, that considerable amounts of ATP are released into the alveolar space following injurious ventilation, which are sufficient to promote an alveolar inflammatory reaction. The efficacy of ATP antagonism in the treatment of VILI should be tested in a clinically-oriented animal model.

## Conclusion

In the present study, we found that extracellular ATP promotes lung inflammation in mice *in vivo*, and that the ATP-P2Y receptor system is involved in the pathogenesis of VILI. The blockade of ATP signaling might, therefore, be a promising treatment of VILI.

## Competing interests

The authors declare that they have no competing interests.

## Authors' contributions

HM performed the experimental studies and drafted the manuscript. FA designed and planned the experiments. SoH, HU, SB and MM assisted with several phases of the study. NS and AI participated in the design of the study. FA and SaH designed the experimental set up, supervised the experimental work, participated in the manuscript preparation and contributed important intellectual content. SaH coordinated the research group. All authors have read and approved the final manuscript.
